# White-Matter Structural Connectivity Underlying Human Laughter-Related Traits Processing

**DOI:** 10.3389/fpsyg.2016.01637

**Published:** 2016-10-27

**Authors:** Ching-Lin Wu, Suyu Zhong, Yu-Chen Chan, Hsueh-Chih Chen, Gaolang Gong, Yong He, Ping Li

**Affiliations:** ^1^Department of Educational Psychology and Counseling, National Taiwan Normal UniversityTaipei, Taiwan; ^2^State Key Laboratory of Cognitive Neuroscience and Learning and IDG/McGovern Institute for Brain Research, Beijing Normal UniversityBeijing, China; ^3^School of Systems Science, Beijing Normal UniversityBeijing, China; ^4^Institute of Learning Sciences, National Tsing Hua UniversityHsinchu, Taiwan; ^5^Department of Psychology and Institute for CyberScience, Pennsylvania State University, University ParkPA, USA

**Keywords:** gelotophobia, diffusion tensor imaging, graph theory, brain network, connectome

## Abstract

Most research into the neural mechanisms of humor has not explicitly focused on the association between emotion and humor on the brain white matter networks mediating this connection. However, this connection is especially salient in gelotophobia (the fear of being laughed at), which is regarded as the presentation of humorlessness, and two related traits, gelotophilia (the enjoyment of being laughed at) and katagelasticism (the enjoyment of laughing at others). Here, we explored whether the topological properties of white matter networks can account for the individual differences in the laughter-related traits of 31 healthy adults. We observed a significant negative correlation between gelotophobia scores and the clustering coefficient, local efficiency and global efficiency, but a positive association between gelotophobia scores and path length in the brain's white matter network. Moreover, the current study revealed that with increasing individual fear of being laughed at, the linking efficiencies in superior frontal gyrus, anterior cingulate cortex, parahippocampal gyrus, and middle temporal gyrus decreased. However, there were no significant correlations between either gelotophilia or katagelasticism scores or the topological properties of the brain white matter network. These findings suggest that the fear of being laughed at is directly related to the level of local and global information processing of the brain network, which might provide new insights into the neural mechanisms of the humor information processing.

## Introduction

Laughter is an innate human emotional expression (Ruch and Ekman, 2001). Different types of laughter have different social consequences: while warm smiles facilitate social interactions, scornful laughing or ridiculing often leaves the target of the laughter having anxiety and pain (Chen et al., 2011). Different people also experience laughter differently: while some people enjoy mocking others, a few enjoy being laughed at. Long-term clinical observations suggest that early and repeated experiences of being mocked and laughed at in childhood and youth are causally associated with the fear of being laughed at, a symptom called gelotophobia (Titze, 2009). Continued experience of gelotophobia may lead to an individual's lack of liveliness, spontaneity, and joy (Titze, 2009). Moreover, individuals with a high gelotophobia score have difficulty perceiving and interpreting their own emotions (Papousek et al., 2009; Weiss et al., 2012); they do not discriminate between playful teasing and nature teasing (Platt, 2008). In addition, individuals with gelotophobia reported experiencing shame and fear more frequently, feeling happy more seldom during a typical week (Platt and Ruch, 2009). Hence, they would less likely enjoy their positive emotions while having high degrees of social anxiety, social phobia, and shame-bound anxiety. EEG research indicated loose functional coupling of prefrontal and posterior cortex in individuals with gelotophobia when expressing anger and aggression (Papousek et al., 2016).

In terms of the personality, gelotophobia positively correlated with introversion and neuroticism and negatively correlated with agreeableness and openness (Ruch et al., 2008; Chen et al., 2011, 2013). Individual with high gelotophobia would be introvert and emotionally instable, while not open to new experiences and be friendly with people.

Ruch et al. (2009) and Ruch and Proyer (2009) examined the psychometric properties of gelotophobia, and identified two related characters and their correlations with gelotophobia: gelotophilia (the enjoyment of being laughed at) and katagelasticism (the enjoyment of laughing at others). So far, research has shown that gelotophobia negatively correlated with gelotophilia but positively associated with katagelasticism (Ruch and Proyer, 2009; Chen et al., 2011).

Previous studies of humor processing have identified the cognitive components of humor processing and their associated neural mechanisms (Ozawa et al., 2000; Goel and Dolan, 2001). In two recent fMRI studies, Chan et al. (2012, 2013) divided the cognitive processes of humor comprehension into three stages: the incongruity stage, which engages the right middle temporal gyrus (MTG) and right medial frontal gyrus (MFG); the solution stage, which is anchored in the left superior frontal gyrus (SFG) and the left inferior parietal lobule (IPL); and the elaboration stage, which includes the left ventromedial prefrontal cortex (vmPFC), the bilateral amygdala, and the bilateral parahippocampal gyri. To date, however, very few studies have specifically examined the emotional functions of humor processing, or applied this knowledge of laughter-related brain networks to investigate the underlying neural mechanisms of laughter-related behavioral traits (i.e., gelotophobia, gelotophilia, and katagelasticism). This is surprising given the observed relationship between gelotophobia and humor processing.

The human brain is a complex network, both structurally and functionally, and consists of densely connected neural units (Sporns et al., 2005; Sporns, 2011). Advances in diffusion imaging techniques have made it possible to clearly delineate white matter (WM) tracts, which allows the modeling of the human brain as a complex network with graph-theoretical analytic tools (Hagmann et al., 2008; Bullmore and Sporns, 2009; Gong et al., 2009). Graph theoretical approaches have been applied effectively to characterize the topological architectures of whole-brain WM networks (Rubinov and Sporns, 2010). Using such approaches, recent studies have made significant progresses in describing the relationship between brain topological characteristics and cognitive function (Li et al., 2009; Wen et al., 2011). Researchers have also begun investigating the relationship between WM structure and individual traits. Specifically, the technique of diffusion tensor imaging (DTI) has enabled researchers to correlate WM structure with aspects of personality, creativity, and other psychological traits (Takeuchi et al., 2010, 2012; Xu and Potenza, 2012). However, no work in this direction that has examined structural or functional brain properties in terms of network models and their potential relationship to laughter-related performances (i.e., gelotophobia, gelotophilia, and katagelasticism). In this study, we attempt to fill this gap in the literature by using DTI and graph-theoretical network analyses.

The present study first attempt to investigate the connection between fear to be laughed at and network topological properties within few direct references. Based on the close relationship between fear to be laughed at and personality (Ruch et al., 2008), we refer to the openness personality positively connect to integrated efficiency of default mode network (Beaty et al., 2016) which includes the dorsolateral and ventral medial prefrontal cortex, posterior cingulate cortex, inferior parietal lobule, lateral temporal cortex, hippocampal formation (Buckner et al., 2008); it means when individual was more opened to new things, and the regions of default mode network worked better, and vice versa.

To address these issues, the present study makes a first attempt to explore the correlations between topological properties of the structural brain network underlying laughter-related traits, including gelotophobia, gelotophilia, and katagelasticism. Previous research has indicated significant relations among the laughter-related traits and personality traits (Chen et al., 2011); gelotophobia positively correlated to neuroticism but was negatively associated to extraversion, agreeableness and openness whereas for gelotophilia, it was positive correlated to extraversion and openness. With regard to the negative relation between katagelasticism and agreeableness. In additions, past research on the relation between five-factor personality (Xu and Potenza, 2012) and white matter integrity also pointed out that, fractional anisotropy was negatively related to neuroticism but positively related to openness and agreeableness. In particular, according to the positive relationship between openness and global efficiency of default mode network (Beaty et al., 2016); we hypothesized that the WM network efficiency would differ significantly between individuals with distinct profiles of gelotophobia, gelotophilia, and katagelasticism characteristics. Given the different characteristics of laughter toward oneself and others, we further expected that the network efficiency would decrease with the fear of being laughed at and the enjoyment of laughing at others, but increase with the enjoyment of being laughed at.

## Methods

### Subjects

Thirty-one neurologically healthy volunteers (18 females; 24.74 ± 2.48 years old; range, 20–30 years old) were included in this study (see Table 1). All participants were recruited from National Taiwan Normal University and had no history of neurological or psychiatric disorders. Participants were asked to refrain from ingesting caffeine and alcohol for the 24 h preceding the experiment. The study was approved by the Research Ethics Committee of National Taiwan University Hospital. All of the subjects gave their written informed consent to participate before the study.

**Table 1:** **Demographic of subjects and PhoPhiKat scores**.

**Category**	**Data**	**Range**
Gender (Male/Female)	13/18	–
Age, years	24.74 ± 2.48	21–30
Education, years	16.06 ± 0.96	14–18
Gelotophobia	2.23 ± 0.42	1.33–3.13
Gelotophilia	2.68 ± 0.42	1.53–3.67
Katagelasticism	2.00 ± 0.39	1.27–2.87

### PhoPhiKat score

Before the experiment, participants completed the Chinese version of the PhoPhiKat-45 (gelotophobia, gelotophilia, and katagelasticism; Chen et al., 2011), a questionnaire translated from the German version (Ruch and Proyer, 2009) that has been successfully used to assess gelotophobia (e.g., “When strangers laugh in my presence I often relate it to me personally”), gelotophilia (e.g., “When I am with other people, I enjoy making jokes at my own expense to make the others laugh.”), and katagelasticism (e.g., I enjoy exposing others and I am happy when they get laughed at). The questionnaire had 45 items, with 15 items devoted to each category of gelotophobia, gelotophilia, and katagelasticism. A 4-point scale was used (1 = “strongly disagree”; 2 = “disagree”; 3 = “agree”; 4 = “strongly agree”) for each item, and the larger the accumulated points under a category, the higher the participant has the corresponding characteristic. The Cronbach α consistency coefficient was 0.85, indicating a high degree of internal consistency. Further, the mild correlations with criterions (i.e., humor-style, aggressive behavior, personality, and self-esteem) were found (*rs* > 0.50), suggesting a moderate validity of the measurement (Chen et al., 2011).

### MRI acquisition

Images were acquired with a 3T scanner (Siemens Trio, Siemens Medical Solutions USA) at National Taiwan University Hospital, Taiwan. DTI were acquired by using a single-shot echo planar imaging-based sequence with sensitivity encoding and the following parameters: a parallel imaging factor of 2.0; coverage of the whole brain; 2.5-mm slice thickness with no inter-slice gap; 60 axial slices; TR = 11,000 ms; TE = 98 ms; 30 optimal non-linear diffusion weighting directions with *b* = 1000 s/mm^2^ and five additional images without diffusion weighting (i.e., *b* = 0 s/mm^2^); average, 3; acquisition matrix, 96 × 96; field of view (FOV), 256 × 248 mm^2^. A T1-weighted MPRAGE sequence was used to acquire high-resolution anatomical images of the entire brain with the following parameters: TR = 1560 ms, TE = 3.68 ms, flip angle = 15⋅, field of view = 256 × 256 mm^2^, and matrix size = 256 × 256; 192 sagittal slices; 1 × 1 × 1 mm^3^ resolution.

### Data preprocessing

The preprocessing pipeline for each subject is composed of the following steps: brain extraction, correction for eddy-current distortion and simple head-motion, correction for b-matrix (Leemans and Jones, 2009), and computation for diffusion tensor and fractional anisotropy. All of the image preprocessing was implemented by a pipeline tool for diffusion MRI (PANDA; Cui et al., 2013) that has utilized the FMRIB Software Library (FSL; Smith et al., 2004), Pipeline System for Octave and Matlab (PSOM; Bellec et al., 2012), Diffusion Toolkit (Wang et al., 2007) and MRIcron (http://www.mccauslandcenter.sc.edu/mricro/mricron/).

### Construction of binary white matter connectivity networks

**Figure 1:**
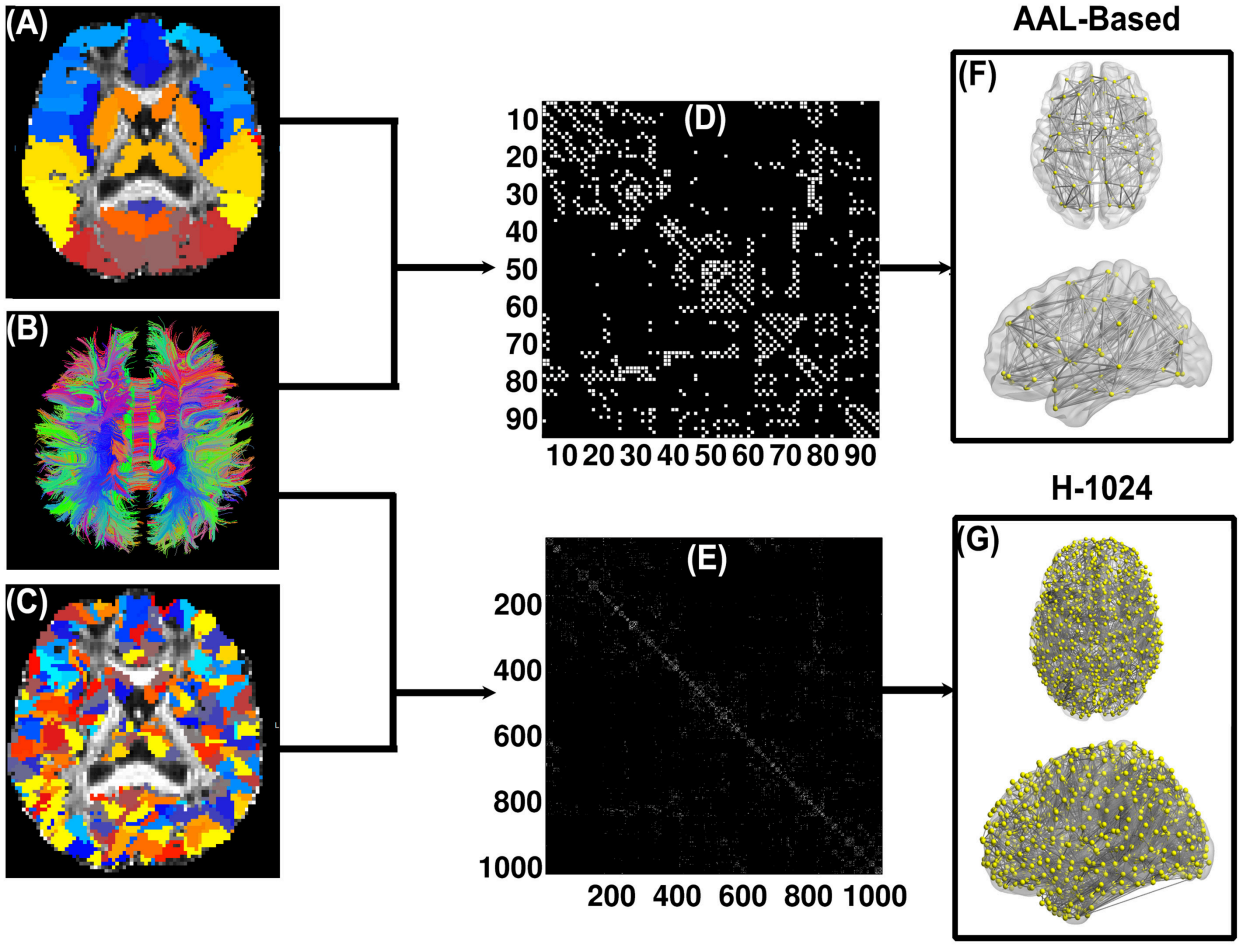
**Flow chart of the DTI-based WM brain network construction**. **(A)** and **(C)** are the subject-specific AAL-based parcellation and H-1024 parcellation in the diffusion native space, respectively; **(B)** shows the white matter fibers reconstructed by deterministic tractography; **(D)** and **(E)** are the AAL-based and H-1024 binary network matrix, respectively; **(F)** and **(G)** are the 3D representation of the anatomical brain network, which was shown using the in-house BrainNet Viewer package (http://www.nitrc.org/projects/bnv/; Xia et al., 2013).

Figure 1 illustrates the flowchart of WM brain network construction. The first step is to determine two basic network elements, nodes and edges, as defined below.

#### Network node definition

In this study, the automated anatomical labeling (AAL, Tzourio-Mazoyer et al., 2002) atlas was used to segment the cerebral cortex of each subject into 90 regions (45 for each hemisphere) without the cerebellum. Each region represents a node of the DTI-based WM network. The detailed parceling processes were implemented according to the procedure proposed by Gong and colleagues (Gong et al., 2009). Briefly, the T1-weighted image was first non-linearly normalized to the MNI space by FMRIB's Linear Image Registration Tool (FNIRT, FSL, http://www.fmrib.ox.ac.uk/fsl/). Next, the fractional anisotropy image of each subject was co-registered to the individual T1-weighted image. Finally, the inverse transformations from the previous two steps were applied to the atlas, resulting in native-space GM parcellations for each subject.

#### Network edge definition

In this study, the deterministic fiber assignment continuous tracking (FACT) algorithm was applied to reconstruct whole-brain WM tracts (Mori et al., 1999) by the Diffusion toolkit (http://trackvis.org), which is embedded in PANDA (Cui et al., 2013). Specifically, the tracking procedure terminated if the turn angle of the fiber was greater than 45⋅ or the fiber entered a voxel with the fractional anisotropy less than 0.2. Two region pairs, A and B, were considered structurally connected (i.e., having an edge) if there existed at least three tracts with terminal points in both regions A and B (Bai et al., 2012). Combining the above definitions of the nodes and edges, we attained for each subject a 90 × 90 binary network whose elements indicated the existence/absence of an edge between any pair-wise regions.

#### High-resolution brain network

Previous research showed that brain graph metrics are dependent on the resolution of the network (i.e., network size) (van den Heuvel et al., 2008; Wang et al., 2009; Zalesky et al., 2010; Bai et al., 2012). To explore the validity of our results, we further subdivided the AAL template into 1024 ROIs with equal size [i.e., high-resolution (H-1024)] (Zalesky et al., 2010; Bai et al., 2012) and constructed the brain networks accordingly. Similar to the low-resolution AAL (L-AAL) networks, the H-1024 networks were examined with respect to the relationships between PhoPhiKat scores and network metrics.

### Network analysis

Graph theoretical measures were used to characterize the topological architecture of the WM brain networks derived above. In the current study, both global network metrics and nodal metrics were computed. The global network metrics were computed for the mean clustering coefficient (*Cp*), the characteristic path length (*Lp*), the normalized *Cp* (γ), the normalized *Lp* (λ), small-worldness (ζ), global efficiency (*E*_*glob*_), and local efficiency (*E*_*loc*_). The nodal network metric was computed only for the nodal efficiency (*E*_*nodal*_). These graph-theoretical network metrics were calculated by using the GRETNA package (http://www.nitrc.org/projects/gretna/).

#### Clustering coefficient *Cp*

The clustering coefficient of a network characterizes the segregation ability of the network by calculating the global mean of the clustering coefficients over all nodes, where the clustering coefficient of a node is defined as the ratio of the number of existing connections among the node's neighbors over all of their possible connections (Bullmore and Sporns, 2009).

#### Characteristic path length *Lp*

The characteristic path length is used to characterize the optimal routing for information transmission. The characteristic path length of a graph refers to the averaged shortest path lengths across all nodes, where the shortest path length of a node *i* is computed as the average number of distinct edges along the shortest path between nodal *i* and all other nodes in the networks. The characteristic path length of a network is computed as follows:

$$L_{p}=\frac{1}{N(N-1)}\sum_{i\in G}\;\sum_{j\in G}\frac{1}{L_{ij}}$$

where *N* is the number of nodes in the graph *G*, and *L*_*ij*_ is the shortest path length between nodes *i* and *j*.

#### Small-worldness ζ

The concept of small-worldness in network science was originally proposed by Watts and Strogatz (Watts and Strogatz, 1998). Specifically, a network is considered a small-world network if it has similar shortest path lengths but higher clustering coefficients than degree-matched random networks. Typically, a small-world network should meet the following criteria: ζ = γ/λ > 1, where γ = *Cp*^*real*^*/Cp*^*random*^ > 1, λ = *Lp*^*real*^*/Lp*^*random*^ ≈1, where *Cp*^*real*^ and *Lp*^*real*^ are clustering coefficient and characteristic path length, respectively, of the real brain network, and *Cp*^*random*^ and *Lp*^*random*^ are the averaged values of the 100 matched random networks, which are the same as the real network in the number of nodes, edges, and degree distribution. The random rewiring procedure depicted by Maslov and Sneppen (Maslov and Sneppen, 2002) was used here to produce the 100 matched random networks for each subject.

#### Global efficiency *E*_*glob*_

Global efficiency is a global measure of the parallel information transfer ability of the whole network. It is computed as the average of the inverse of the “harmonic mean” of the characteristic path length (Latora and Marchiori, 2001):

$$E_{glob}=\frac{1}{N(N-1)}\sum_{i\neq j\in G}\frac{1}{L_{ij}}$$

where *N* is the number of nodes in the graph *G*, and *L*_*ij*_ is the shortest path length between nodes *i* and *j*.

#### Local efficiency *E*_*loc*_

Local efficiency quantifies the network's ability to tolerate faults, corresponding to the efficiency of the information flow between the nearest neighbors of the node *i* (Latora and Marchiori, 2001). The local efficiency of a network is computed as follows:

$$E_{loc}=\frac{1}{N}\sum_{i\in G}E_{glob}(G_{i})$$

where *G*_*i*_ is the sub-graph composed of the nearest neighbors of node *i* and the connections among them.

#### Nodal efficiency *E*_*nodal*_

Nodal efficiency is a measure of the nodal capacity to communicate with other nodes of the network. The nodal efficiency for a given node (*E*_*nodal*_) was defined as the inverse of the harmonic mean of the shortest path length between this node and all other nodes in the network (Achard and Bullmore, 2007):

$$E_{nodal}(i)=\frac{1}{N-1}\sum_{i\neq j\in G}\frac{1}{L_{ij}}$$

where *L*_*ij*_ is the characteristic path length between node *i* and node *j*.

### Statistical analysis

To explore the relationship between the topological parameters (*Cp, Lp*, ζ, *E*_*loc*_*, E*_*glob*_*, and E*_*nodal*_) of WM brain networks and PhoPhiKat scores, general linear models (GLM) were applied with age, gender and years of education as covariates. Specifically, the GLM is as follows: Y = β_0_ + β_1_ × X + β_2_ × Age + β_3_ × Gender + β_4_ × Education, where Y is the topological parameter and X the PhoPhiKat score. The correlation was determined by examining the null hypothesis of β_1_ = 0. The threshold value for establishing the significance of correlation was set at *p* < 0.05 for the global metrics, *p* < 1/N (corrected for multiple comparisons) for the nodal metrics of the AAL-based networks.

## Results

### Small-world properties of brain networks

The structural brain networks of all subjects showed a small-world architecture. More specifically, when compared with a matched random network, the WM networks had comparably shortest path lengths but higher clustering coefficients (ζ = 3.54 ± 0.32), suggesting that the overall topological properties were preserved regardless of the scores from the scales for gelotophobia, gelotophilia, and katagelasticism.

### PhoPhiKat scores and network properties

**Figure 2:**
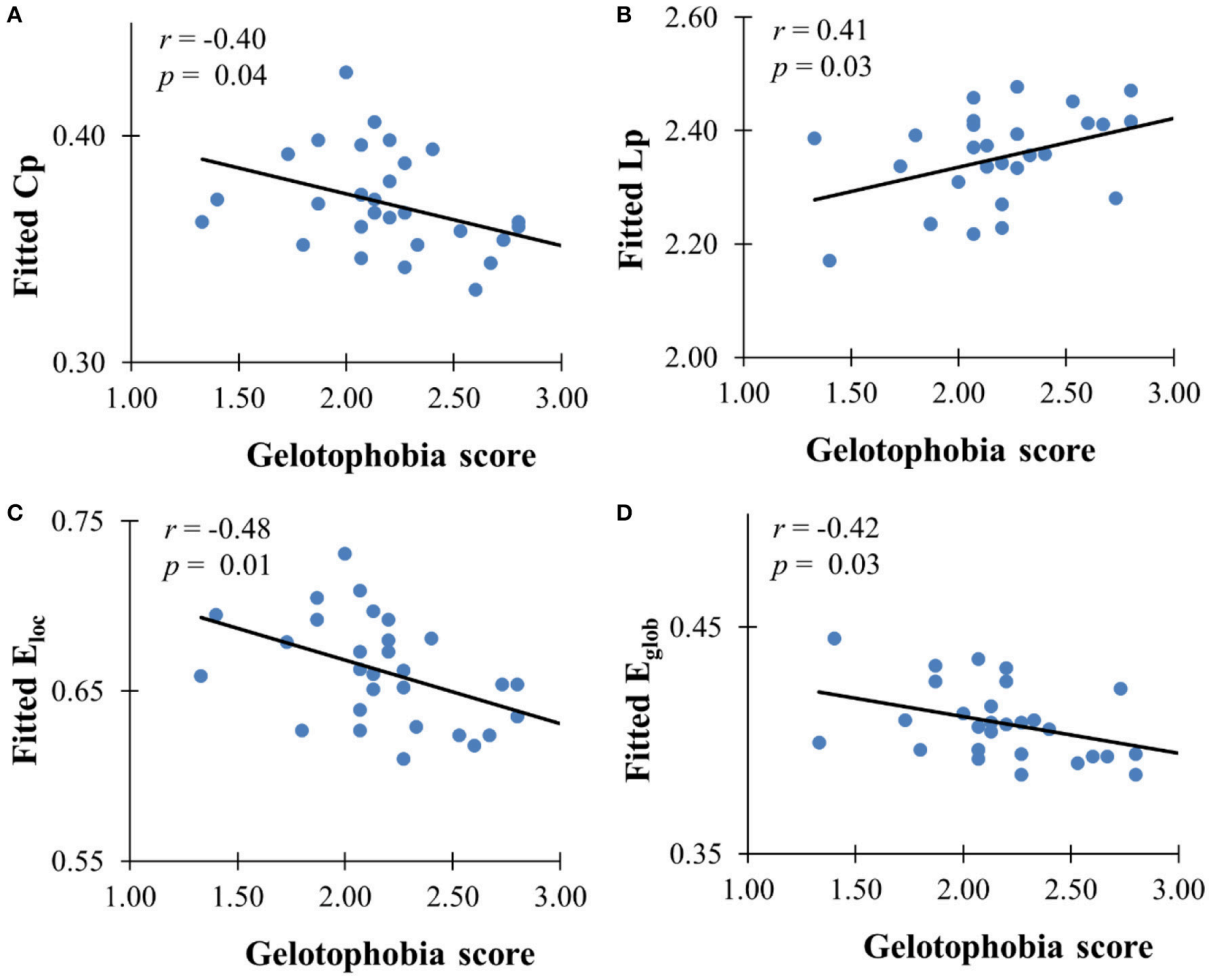
**The correlation between gelotophobia score and global metrics derived from AAL-based WM network**. **(A)** Clustering coefficient, **(B)** path length, **(C)** local efficiency, and **(D)** global efficiency. For all plots, we used linear regression to remove the influence of age, gender and years of education from brain network metrics.

The GLM analysis showed a significant negative association between gelotophobia scores and the clustering coefficient (*r* = −0.40, *p* = 0.037), local efficiency (*r* = −0.48, *p* = 0.01), and global efficiency (*r* = −0.42, *p* = 0.027) after controlling for age, gender, and years of education, and a significant positive association with the characteristic path length (*r* = 0.41, *p* = 0.029, see Figure 2). However, there were no significant correlations between gelotophilia or katagelasticism scores and the topological properties of the WM network.

**Figure 3:**
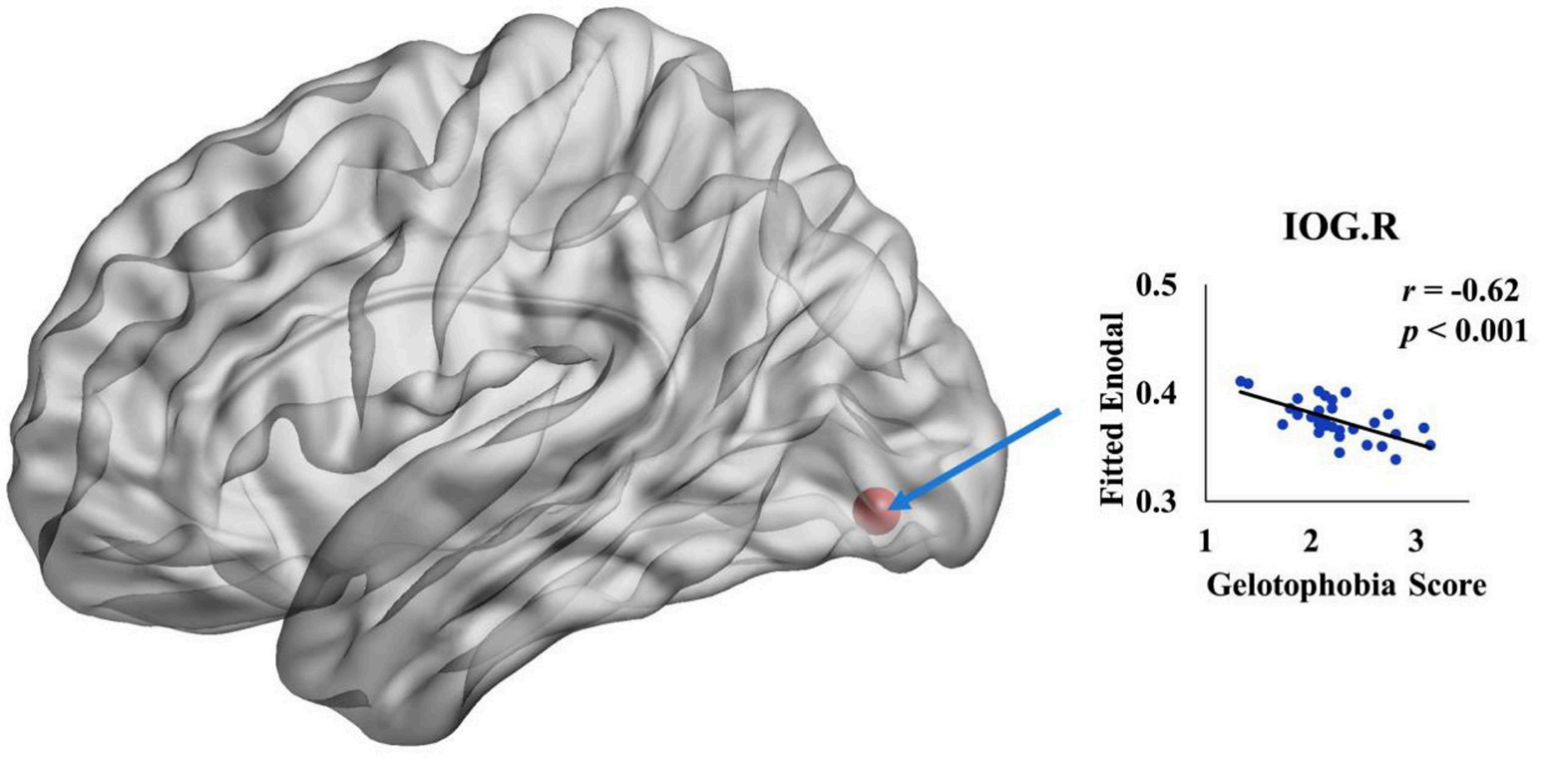
**The spatial distribution of cortical region showing a significant association between nodal efficiency in the right inferior occipital gyrus and gelotophobia score (*p* < 0.05/90)**.

We further investigated the specific brain regions associated with the tendency of an individual to fear being laughed at. Figure 3 shows the 3D surface visualizations of the results implemented using the Brain Net Viewer (www.nitrc.org/projects/bnv; Xia et al., 2013). As can be seen, significant correlations between gelotophobia scores and nodal efficiency were found in the inferior occipital gyrus (*r* = −0.62, *p* < 0.0001). Notably, we corrected for age, gender and years of education when computing the correlation, and we used *p* < 0.05/90 to correct for multiple comparisons.

#### High-resolution structural brain networks

**Figure 4:**
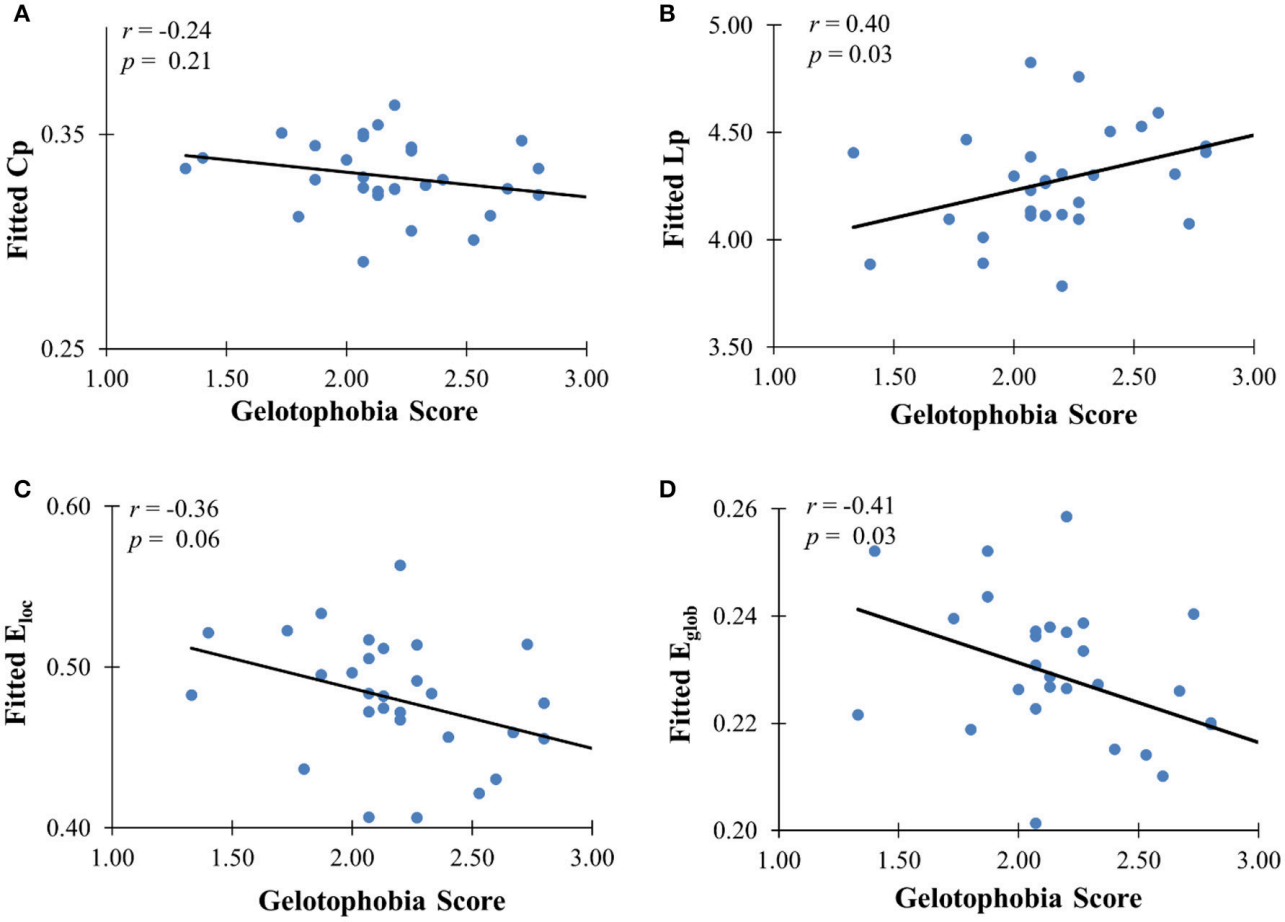
**The correlation between gelotophobia score and H-1024 WM network metrics. (A)** Clustering coefficient, **(B)** path length, **(C)** local efficiency, and **(D)** global efficiency; For all plots, we used linear regression to remove the influence of age, gender and years of education from brain network metrics.

**Figure 5:**
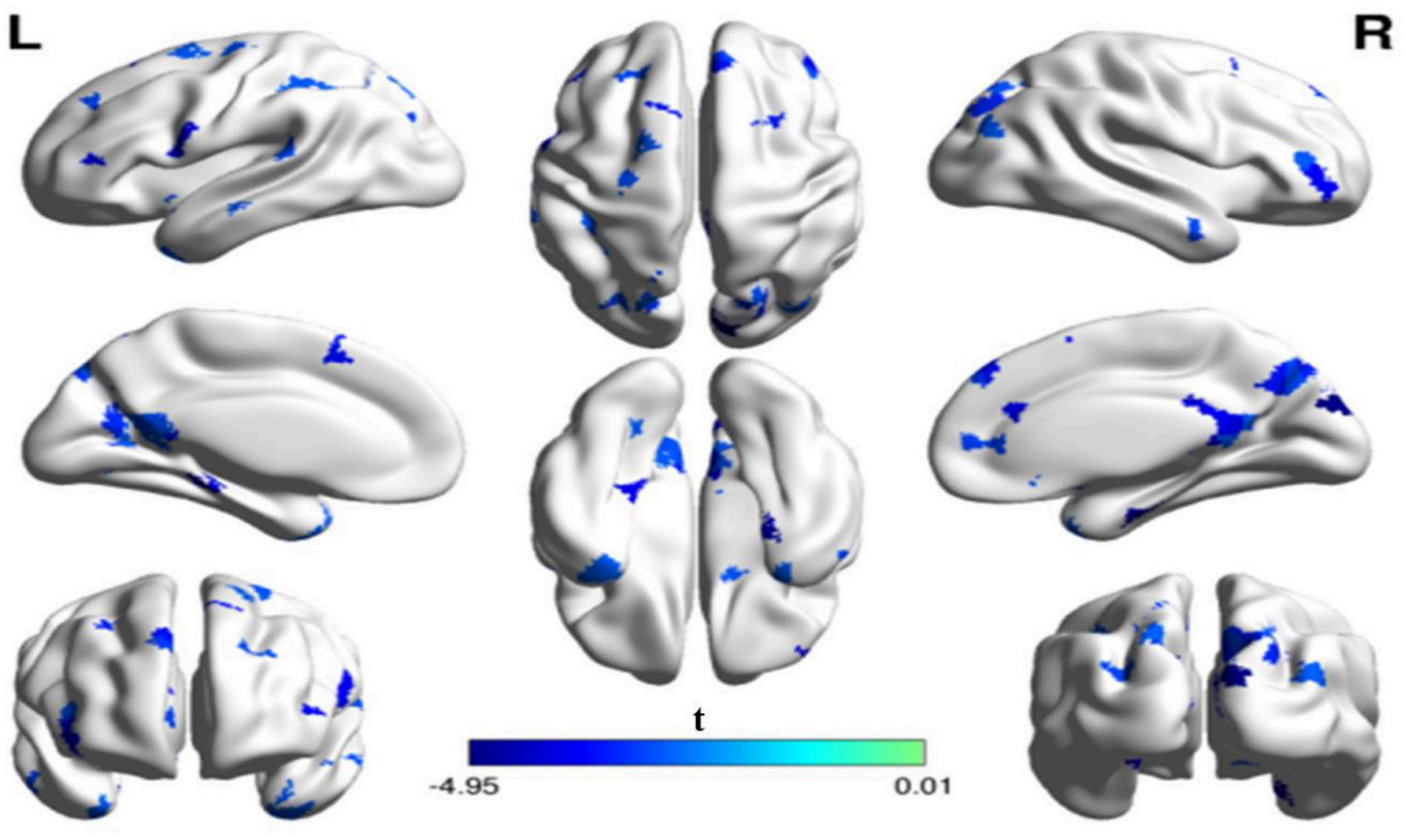
**The spatial distribution of cortical regions showing a significant association between nodal efficiency and gelotophobia score (*p* < 0.01, uncorrected) in an H-1024 network**. Note that three regions (the ACC, PHG, MTG) appeared in a bilateral manner and the CUN. R, right hemisphere; ACC, anterior cingulate cortex; CUN, Cuneus; PHG, parahippocampal gyrus; MTG, middle temporal gyrus.

In addition to the analyses of AAL-based networks, we further analyzed the relationship between the high resolution-based H-1024 network and the PhoPhiKat scores. Consistent with findings from the AAL-based networks, there were no relationships between gelotophilia or katagelasticism and brain network properties, but there were significant negative associations between gelotophobia scores and local efficiency (*r* = −0.36, one-tailed *p* = 0.03) and global efficiency (*r* = −0.41, *p* = 0.028; see Figure 4). As shown in Figure 5, significant correlation between the nodal efficiency and gelotophobia score was found in right cuneus (*r* = −0.70, *p* < 0.001, uncorrected), parahippocampal gyrus (*r* = −0.67, *p* < 0.001, uncorrected), anterior cingulate and paracingulate gyri (*r* = −0.61, *p* < 0.001, uncorrected), and middle temporal gyrus (*r* = −0.50, *p* = 0.007, uncorrected). Furthermore, a significant positive association was also found between the characteristic path length and gelotophobia score (*r* = 0.41, *p* = 0.032). The only difference between the high resolution-based networks and the AAL-based networks seems to be that gelotophobia score showed no correlation with the clustering coefficient (*r* = −0.24, *p* = 0.214).

## Discussion

Gelotophobia refers to the experience of intense fear felt by an individual when being laughed at (Titze, 1996, 1997). Gelotophobia can cause significant problems in people's lives, leading them to be socially inept, cold, mean-spirited, or depressed. However, the neural mechanisms underlying gelotophobia are not well-understood, as there has been no research designed to address this question. The present study is a first attempt to uncover the neuro-cognitive mechanisms underlying gelotophobia and related laughter-related traits from the perspective of brain networks. Specifically, we aim at understanding how WM structure in the human brain subserves human laughter-related processing, given the role of WM in facilitating neural processing and communication across areas of the cortex and between cortical and subcortical regions.

The main finding of this study is the existence of a strong association between the topological metrics of the WM brain network and gelotophobia scores at both the global and the nodal level. For the global metrics, our results revealed a significant negative association between gelotophobia scores and the clustering coefficient, local efficiency and global efficiency; for the nodal metric, we observed a strong positive association between gelotophobia scores and the path length of the structural brain networks. Given that both global and local efficiency metrics significantly and negatively associated with gelotophobia scores, we suggest that an individual's fear of being laughed at related to the level of local and global information processing of the brain network. In other words, less efficiently connected brain structures show a higher level of gelotophobia. Part of our finding is also supported by a further analysis with High-resolution structural brain networks. Present study is a first attempt for discussing the connection between fear to be laughed at and WM network topological properties. Individual with fear to be laughed at had higher hostility and less stable emotion status, less interest to new things (Ruch et al., 2008). Our results are similar with the findings between openness personality and integrated efficiency of default mode network. Although the AAL network that we use is different from default mode network, but the finding still supports a decrease global efficiency of WM network among individual with gelotophobia which would not intend to open to new experience.

In addition to the overall correlations between brain network metrics and the gelotophobia score, our data also revealed that the more an individual fears being laughed at, the lower the connecting efficiencies the individual displays in right inferior occipital gyrus (IOG). IOG is relevant to emotions recognition; functional MRI result indicated a higher activation in right IOG during the process of identifying an anger face (Fusar-Poli et al., 2009). On the other hand, DTI results found a positive correlation between the mean diffusion (MD) of inferior frontal occipital fasciculusçŽĎmean diffusion (MD) and openness (Xu and Potenza, 2012). In particular, individuals with fear to be laughed at had less interest to trying something new (Chen et al., 2011), it was consistent to the finding that IOG and fear to be laughed at had negative connection. Although EEG research result could not provide detail information of brain regions, but it indicated the weakness of brain function for people with fear to be laughed at, which supports our finding, a negative connection between nodal efficiency and trait of fear to be laughed at. However, present study did not collect data about personality of participants; we could not investigate moderate effect of personality between fear to be laughed at and brain network. It's worthwhile for further study.

The present findings suggest that gelotophilia and katagelasticism were not significantly correlated with nodal efficiency of the WM network. It is possible that clearly defined neural networks exist only for gelotophobia because this condition was defined based upon rigorous long-term observations among patients by Titze (2009) and was a clinical syndrome concluded by qualitative analysis. In contrast, gelotophilia and katagelasticism were developed as expanded and ancillary concepts by theoretical generalizations about gelotophobia. The correlations between gelotophobia and mental traits are much stronger than the ones between gelotophilia, katagelasticism and mental traits (Ruch and Proyer, 2009; Chen et al., 2011); thus, significant associations between topological properties of the WM network and gelotophilia or katagelasticism could not be found.

There remain two methodological issues that we should consider for future follow-up research. First, we used the number of fibers as the weighting factor in the construction of the graphs, rather than other measures such as average fractional anisotropy (FA), mean diffusivity (MD), or a combination of such measures. FA and MD are measures of different aspects of the fibers; e.g., FA is relevant to fiber “integrity (magnitude of diffusion of water molecules in the brain)” whereas number of fibers is relevant to fiber “quantity.” Whether analyses based on FA, MD, and similar measures will provide similar brain network patterns as we showed in this study needs further investigation. Second, we built two spatial resolutions of the WM network in the present study, L-AAL and H-1024, to analyze the relationship between typological properties of the networks and the PhoPhiKat laughter-processing scores. There were some discrepancies in the regions that were correlated with gelotophobia between the L-AAL and H-1024 WM networks. Such discrepancies may be moderated by differences in the graph properties under the subregions of some anatomical structures. It revealed that constructing high-resolution WM network can provide a useful way to support or validate L-AAL-based WM network analyses. Therefore, graph analyses with different spatial resolutions in the future should be conducted with an aim to provide a more comprehensive picture of the topological properties of brain networks in the clinically normal and disordered populations.

## Conclusion

The present study revealed significant correlations between gelotophobia and the underlying organization of the cortical anatomical network within the superior frontal gyrus, anterior cingulate cortex, parahippocampal gyrus, and middle temporal gyrus. Our study provides new insights into the structural substrates that underlie the individual personality trait of fear of being laughed at. These findings reveal patterns and efficiency of brain networks in gelotophobic individuals and at the same time provide significant implications for evaluating clinical populations with reference to altered connectivity in the neuroanatomy of the relevant populations.

## Author contributions

C-LW and SZ collected and analyzed the data and wrote the initial draft of the manuscript. Y-CC assisted in literature review and discussion. H-CC designed this study. YH, GG, and PL monitored and supervised all aspects of the study. All authors approved the final version of the paper.

### Conflict of interest statement

The authors declare that the research was conducted in the absence of any commercial or financial relationships that could be construed as a potential conflict of interest.
